# Global declarations on electric vehicles, carbon life cycle and Nash equilibrium

**DOI:** 10.1007/s10098-022-02399-7

**Published:** 2022-09-19

**Authors:** Baher Bakhtyar, Zhang Qi, Muhammad Azam, Salim Rashid

**Affiliations:** 1 Department Business and Economics USIC, University of Sheffield, Sheffield, UK; 2 Department of Economics, Faculty of Business and Economics, Abdul Wali Khan University Mardan, Khyber Pakhtunkhwa, Pakistan; 3 Department of Economics, University of Illinois, Champaign, IL USA

**Keywords:** Electric vehicle, Carbon life cycle, Universal declaration, Carbon emission, Electricity generation

## Abstract

Universal environmental policies adopt strategies that enhance and encourage the production and usage of electric vehicles (EVs). Universal cooperation is evident in the framework of agreements or protocols so as to successfully lead countries towards the predetermined goals. The question is whether this trend can reduce global warming or CO_2_ emissions worldwide. By adopting game theory, this study analyses electricity carbon life cycle in leading EV countries. Results show that although the spread of EVs in Europe and the USA can mitigate carbon emissions, the production and use of electric vehicles in some countries, such as China and India, become a new source of such emissions. This reverse effect is due to the emission of greenhouse gases from electricity sources in these countries. Game theory also suggests that countries with unclean electricity sources should reconsider their plans to produce and use EVs. This study confirms that although carbon emission and global warming are global problems, regional and local policies can be substituted with a single comprehensive approach for an effective means of CO_2_ emission reduction.

**Graphical abstract:**

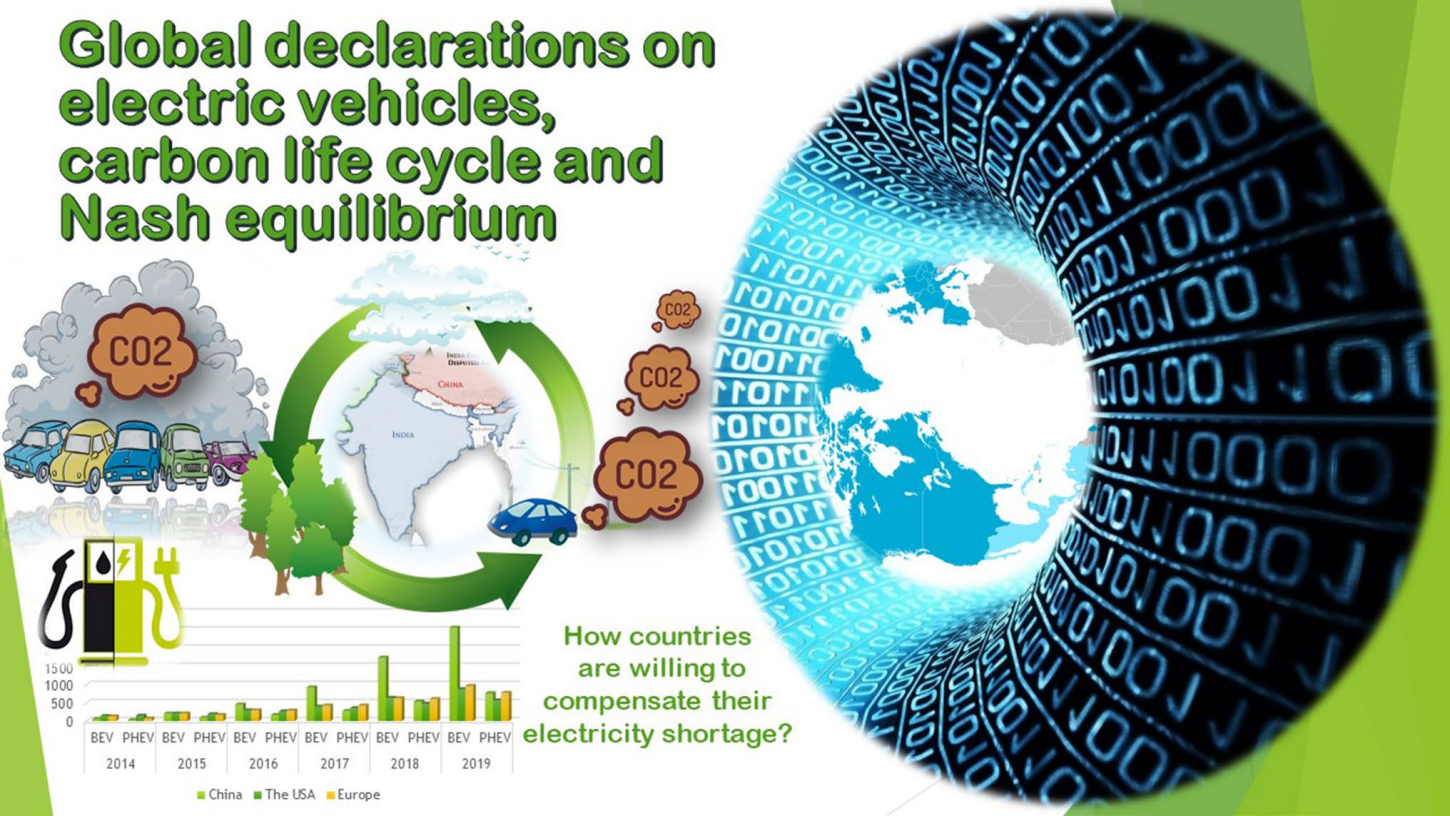

## Introduction

The transition from petroleum as an energy source in the transportation sector and concerns over the climate change issues result in increasing numbers of electric vehicles (EVs) being included in many transport plans of governments around the world (Needell et al. 2016). A year after the Paris Agreement was enacted in 2015, governments confirmed that the constructive spirits of multilateral cooperation on climate change continue at the Morocco Climate Change Conference 2016. Eight major countries, namely Canada, China, France, Japan, Norway, Sweden, the UK and the USA, vowed to increase the percentage of EVs in their government fleets (IEA 2022). These countries also pledged to encourage other countries to join the pioneers.

Decisions adopted in Morocco include the support of the policies of the eight major countries in establishing various incentives for the use of electric cars (Duffy and Opp 2017). In 2017, the United Nations (UN) Climate Change Conference discussed previous agreements on climate change despite the shock of the USA. Finally, a group of 30 countries introduced the Powering Past Coal Alliance (PPCA), which targets power generation without coal in 2030 (Hurri 2020). After the USA left, China got the leading role in most of the panels at Katowice (2018), and the number of country members of PPCA increased to 80 countries in this conference (Blondeel et al. 2020). In 2019 in Madrid, although major carbon producer countries blocked the way of any agreement, the European Union (EU) agreed on the European Green New Deal, which aims zero emission by 2050 (Davidson 2019). Although the coronavirus disease 2019 (COVID-19) halted many climate activities in 2020, including UN conferences that were planned to be held in Glasgow, it was the reason to intensively decrease carbon emissions and improve the quality of climate all over the world, at least for a limited time (Gabbatiss 2020; Kroll et al. 2020).

The Cop26 summit was finally held in Glasgow in November 2021, and the countries agreed on a statement aimed at limiting global warming to 1.5°C. At the last moment, the Indian representative opposed the term ‘phasing out’ coal, and the summit replaced it with ‘phasing down’ in the final statement (Guardian 2021).

In line with the policies adopted at the UN Climate Change Conferences, various new policies are published in the USA, China and European countries as the major producers and markets to achieve the intended objectives. In 2017, the European Commission announced a new restriction on carbon emissions for carmakers (Hoppe and Kersting 2017). Apart from France and Britain 2045, Netherland 2035 and Norway 2025 aim to ban gas and diesel in their countries (Hoppe and Kersting 2017; Figueres et al. 2018). In November 2020, Britain pushed forward a ban on non-hybrid fossil cars in 2030 (Guardian 2021). The European Commission planned Electric Vehicle Quotas for the years after 2021 in attempt to regulate automobile users on the basis of g/km CO_2_ emissions (Hoppe and Kersting 2017).

The most successful US plan for EVs is called the Zero Emission Vehicle (ZEV) programme. The ZEV programme is a set of regulations which push carmakers to sell EVs in California, Colorado, Connecticut, Maine, Maryland, Massachusetts, New Jersey, New York, Oregon, Rhode Island and Vermont (Milovanoff 2020). The main target of this programme is to ensure EV makers, researchers and developers by expanding the market with more than 40 zero emission models available to the US public (Axsen et al. 2020; UCS 2017).

China introduced various programmes, such as Accelerating New Energy Vehicles Promotion (2014), Financial Support Scheme for New Energy Vehicle Promotion 2016–2020, Double Quota Programme (Zhang and Qin 2018)—CAFC and NEV quotas (2019) and Electric Vehicle Charging Infrastructure Development 2015–2020 (Wang et al. 2018; Du et al. 2018). These policies support electric car production and market in general. According to some new rules, EVs are eligible to exemption from traffic regulation and support for promoting charging infrastructure (Li et al. 2018; Ji and Huang 2018).

**Fig. 1:**
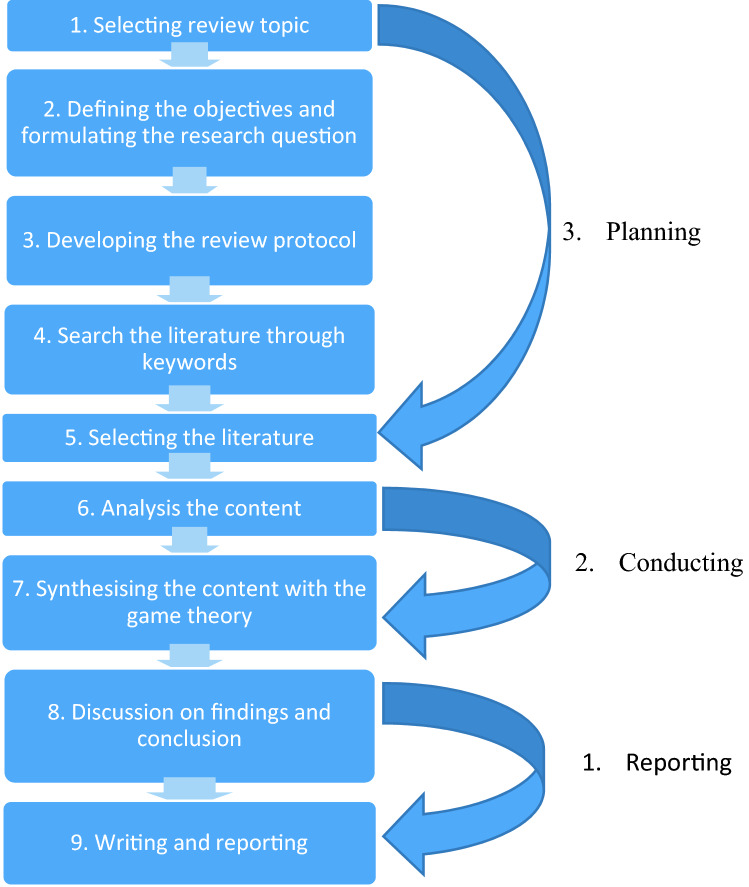
Key steps in conducting narrative review in this study

This work is based on a narrative approach (Fig. 1), which starts with studying the universal declarations and analysing major EV countries. Figure 1 shows the Key steps in conducting the study.

In this study, the major EV countries are those that are leading EV production and usage in the world. The authors classified European countries as a unique leading district as all European countries follow the same EU rule. The USA is another leading country, despite all variations in regulations and EV production in different states. China, as the most populated country, has an aggressive plan towards electric transportations. Finally, the authors look at India because this country expected to be a leading EV country in the next decade.

This study is based on the diffusion of CO_2_ during electricity generation and EV manufacturing and intends to address one of the weaknesses of universal declarations. Countries have different potentials and capacities, and a single policy for all regions and countries of the world can create problems or has low efficiency. For example, banning the use of coal in developed European countries does not have the same consequences in countries such as China and India because it seriously disrupts employment and growth and development of the countries.

This study shifts the view of global organisations and policymakers from ideal conditions to real situations, ultimately leading to more realistic universal declarations. This kind of view of the region’s existing resources and economic conditions leads to the improvement of the initial drafts of the global statements and eventually to the advancement of the efficiency of the major policies of the international organisations. The EV in this study is an example of this type of public prescriptions for various countries. This study challenges the performance of universal declarations in the fight against air pollution and global warming and shows why the failure of some targets can be predicted.

### EV trend and global carbon emission

The question is whether the trend in producing and using electric cars can reduce carbon emission worldwide and result in a decline in global warming. In addition to building EVs, electricity is required for their use. Most of these cars are charged at night and by residential electricity, which, in turn, increases the power consumption of homes. Fischer et al. (2019) confirmed that load peaks in residential electricity usage strongly depend on the deployed charging infrastructure and can easily increase by up to 3.6 times than the present number (Fischer et al. 2019). For high-power charger systems, countries need various charging profiles and can make new grid distribution problems (Sharma and Sharma 2019). The main question here is how willing are countries to compensate their electricity shortage. This question also highlights that selected countries should determine which type of electricity sources are/will develop their EVs.

Consequently, authors analyse electricity resources for the selected areas based on their electricity carbon life cycle calculation. Published reports about electricity sources in some of the leading EV countries confirm that these countries burn coals to generate electricity for EV usage and even EV production in their factories (Chen et al. 2018; Pehl et al. 2017; Oberschelp et al. 2019).

### Clean EV versus polluter EV

Recent claims suggest that electric cars do not cause any pollution and that the zero emission goal can be achieved by increasing the production and use of electric cars (IEA 2017). However, researchers know that only by considering specific conditions in the production and electricity sources of EV can countries reduce carbon emissions. Discussing how to achieve the zero emission goal in the transportation sector or automotive industry is too early (Casals et al. 2016). No convincing evidence shows that EVs are produced as a substitute for fossil fuel cars in the universal scale. The fuel car market is growing (Helmerset al. 2019); the EV market is also growing (Bloomberg 2019) in parallel but not to replace petrol or diesel cars. Thus, the production and deployment of EVs should be closely studied.

Several case studies confirm that the manufacturing of EVs has between 15% and 68% higher emissions than that of normal petrol cars (Janjic and Petrusic 2014; Archsmith et al. 2015; OAM 2012). However, the amount of emitted carbon is subjective and dependent on energy sources. An EV produced in Kentucky, where 73% of electricity comes from coal (EIA-Kentucky 2020) produces more carbon emission than a similar EV manufactured in Alaska, with 47% gas and 27% hydroelectric power (2020) as energy sources (EIA-Alaska 2020).

In the global scale, the scenario is the same. EV production in France, where the major energy source is nuclear power, is not significantly different from normal petrol car production, compared with EV production in countries where the main energy source is coal or heavy oil. These countries emit a large amount of CO_2_ in the manufacturing of each electric car compared with that of similarly sized petrol cars. In general, manufacturing emission can vary up to 30% depending on the variety of energy sources used in different factories or countries (Edelstein 2016).

Conventional plants generate electricity using oil, coal and natural gas. EV owners must acknowledge their share in global warming because the CO_2_ production of these cars is largely subjected to electricity sources. The life cycle estimates of electricity generation from coal, oil/diesel and natural gas produce 1050, 778 and 443 g CO_2_e/kWh, respectively (Sovacool 2008). These estimates indicate that an EV consuming electricity with natural gas as the primary source produces less pollution than the same EV that uses electricity from coal.

The EV emissions by country on the basis of the EV life cycle are measured in g CO_2_e/km and determine the total emissions for EVs, including the total emissions for EVs and manufacture emissions; CO_2_ emissions from fuel combustion in power plants; CO_2_, N_2_O and CH_4_ emissions from fuel extraction, transportation, processing, distribution and storage; and grid losses. Accordingly, the ranges of emission are 70 for Iceland and Paraguay, 318 for South Africa and 370 for India (Wilson 2013). Furthermore, EVs in Paraguay represent a clean, anti-global warming industry, whereas the transportation facility in India produces 370 g CO_2_/km that has more pollution than normal petrol cars.

For instance, a research shows that a petrol Toyota Corolla 1.8 (made in 2012) produces 157 g CO_2_/km; EVs in India, South Africa, Australia, Indonesia and China produce 370, 318, 292, 270 and 258 g CO_2_e/km, respectively (Stuart 2012). A research by University of Sydney (2019) confirms that in many countries, hybrid cars have less emission than electric and conventional cars and EVs cannot be the best choice. For example, a small Toyota Corolla in Australia (2L 4cyl Petrol 91RON, 1 Spd CVT, 4-door 5-seat Hatch, 2WD, Released: 2018) has 163 g CO_2_e/km, whereas the same size electric BNMi3 (BMW I01 i3 i3s BEV 120Ah Pure Electric, 1 Spd Other, 4-door 4-seat Sedan, 2WD, Released: 2019) has 130 g CO_2_e/km. However, a hybrid Toyota Corolla (1.8L 4cyl Electric/Petrol 91RON, 1 Spd CVT, 4-door 5-seat Hatch, 2WD, Released: 2018) has only 101 g CO_2_e/km (UoS 2019).

As previously mentioned, EV emissions depend largely on the variety of electricity sources in different geographic locations. The average CO_2_ emission per 1 kWh of generated electricity in Canada, Japan, Australia and South Africa are 220, 505, 752 and 949 g CO_2_/kWh, respectively (Bakhtyar et al. 2014). The amounts for the UK, Italy, Germany, Greece and France are 511, 443, 556, 732 and 112 g CO_2_/kWh, respectively (Bakhtyar et al. 2017).

### EV market

More than 2,264,000 plug-in vehicles were sold worldwide in 2019, which shows a 9% increase compared with that in 2018 (Irle 2020). Experts believe that market development in the two largest economies (China and Europe) pushed EV market higher than expected in 2018 and 2019 (Jones et al. 2020). Rationally, 2020 is excluded in our survey because of COVID-19. By the end of 2019, the stock of light-duty plug-in vehicles totalled approximately 7.5 million units and more than 700,000 plug-ins were added to the world’s EV sales (ZER 2016). In 2018, the number of electric passenger cars with a 63% increase passed 5,000,000 units.

The worldwide sales including battery electric vehicles (BEVs) and plug-in hybrid electric vehicles (PHEVs) show that China, the USA and Norway are on the top of the list in 2019. China ranked first by selling more than 3,367,000 cars in 2019, whereas the USA sold 1,448,000 cars. Norway, as the top European country, sold more than 384,000 new cars which are more than those sold in Germany and France (Irle 2020).

In 2008, China, as the most populated country, started providing various incentives, including subsidies, cash payments, no-license driving and tax exemptions (Wang et al. 2017). After President Xi Jinping called for an ‘energy revolution’ in 2014, the production and sale of electric cars accelerated. As a result of government support and incentives, China’s electric car sales increased by 223% in 2015 and 188% in July 2016, thus overtaking the USA (Zhaoyuan and Ishwaran 2020).

The cumulative sales in September 2016 were approximately 570,000 EVs in Europe, 521,403 in the USA, 521,649 in China and 145,000 in Japan (Cobb 2016). Furthermore, the order changed rapidly as China’s large market showed a tendency towards EVs, following the government support.

In 2016, China became the largest plug-in electric bus market in the world with a stock of 173,000 EVs (ZER 2016). In 2017, China’s market sold 930,000 BEVs and 280,000 PHEVs. In 2018, this amount increased to 1,750,000 BEVs and 540,000 PHEVs. By 2019, the amount grew to 2,580,000 BEVs and 770,000 PHEVs (IEA 2020), indicating a 277% increase in EV sales in China between 2017 and 2019, as presented in Table 1.

**Table 1:** BEV and PHEV sales in major EV districts (IEA 2020)

Major dealers	2014		2015		2016		2017		2018		2019	
	BEV	PHEV	BEV	PHEV	BEV	PHEV	BEV	PHEV	BEV	PHEV	BEV	PHEV
China	60	30	210	90	460	170	930	280	1750	540	2580	770
USA	140	150	210	190	300	270	400	360	640	480	880	570
Europe	130	70	210	170	300	290	430	430	630	610	970	780

The numbers denote thousand vehicles

China’s EV market is not limited to electric cars as it continues to lead in electrifying two-/three-wheeler vehicles and urban buses. Almost 500,000 buses are in world circulation, and most of them are in China. In 2019, approximately 100,000 unit buses were delivered globally. Approximately 95% of these global deliveries belonged to China (Irle 2020). Although the electric bus market declined between 2016 and 2020, in 2019, China registered 72,000 new E-buses more than Europe (2000 registered E-buses) and other parts of the world (1440 registered E-buses) (IEA 2020; Zhaoyuan and Ishwaran 2020).

Although the USA is proud to be the revival place of EVs, the Americans now seem to be lagging behind their Chinese and European rivals. As exhibited at the present time, the US market is not as rapid as the Chinese market, and the one million EVs targeted by the US president for 2015 was not reached (Sheperdson 2016). The low prices of diesel and petrol may be the main barrier to reach the 2020 target (De Rubens 2018).

**Fig. 2:**
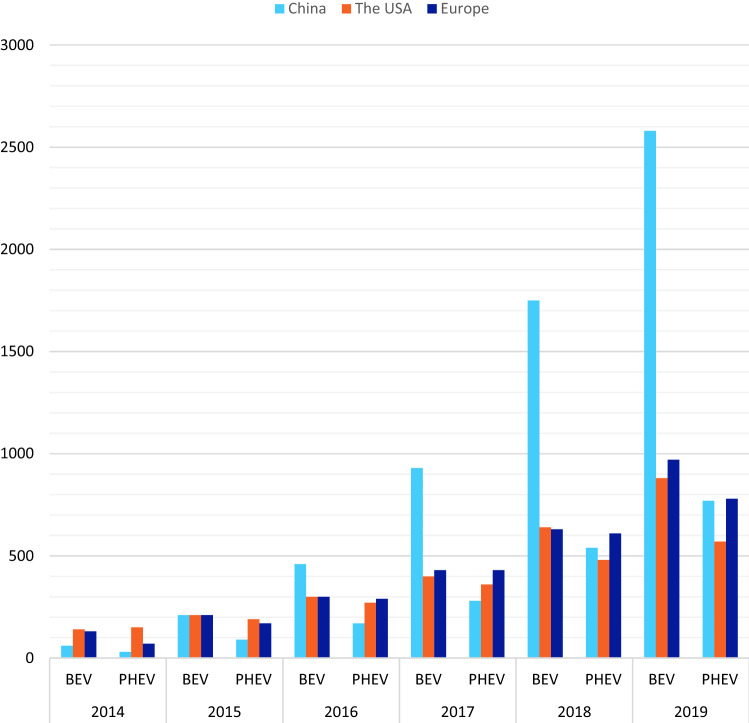
BEV and PHEV markets in leading EV countries (2014–2019)

The US EV market sold 210,000 BEVs and 190,000 BHEVs in 2015. The following year, the market traded 300,000 BEVs and 270,000 PHEVs. The amounts of BEV and PHEV increased to 400,000 and 360,000 in 2017, respectively. The US EV market showed a stable development and vended 640,000 BEVs and 480,000 PHEVs in 2018 and 880,000 BEVs and 570,000 PHEVs in 2019 (IEA 2020; Zhaoyuan and Ishwaran 2020). Figure 2 indicates the BEV and PHEV production trends in China, the USA and Europe between 2014 and 2019.

As presented in Figure 2, Europe has an increasing number of EV fleets in BEVs and PHEVs. The European market confirms that the EV market has been seriously accelerating since 2014 when Europeans were able to deal 130,000 BEVs and 70,000 BHEVs. The European market passed the US market in 2016 in BEV and BHEV production for the first time (IEA 2020). Although a tight competition exists in BHEV, China seems unattainable in the BEV market. In 2019, China sold more BEVs than Europe and the USA when they had an equal share in 2015.

In recent years, other competitors have also entered the EV market, such as India. India has an ambitious policy that aims to be a 100% EV country by 2030 (BBC 2019). It started using 530 EVs in 2009. This number increased to 4,350 in 2015. The National Electric Mobility Mission 2020 is helping India emerge as a leader in the affordable and efficient two-wheeler and four-wheeler EV market in the world by 2020 (Dixit 2020; Sarode and Sarode 2020). Similar to China, India has an aggressive approach to the EV market (Mohanty and Kotak 2017). The high population and large market sizes in both countries have motivated EV companies to help in advancing their respective markets.

### Electricity carbon life cycle in major EV countries

One of the most practical and most comfortable ways to enter the EV market is finding the average CO_2_ emission per kWh of generated electricity in a particular country. The electricity carbon life cycle shows the amount of carbon dioxide released for each kWh of electricity.

Sovacool (2008) collected the data of carbon dioxide ranges, as shown in Table 2. The table indicates the estimated (g CO_2_e/kWh) for common energy sources, including fossil or renewable energy sources.

**Table 2:** Carbon life cycle estimates for selected electricity resources (Sovacool 2008)

Technology	Capacity/configuration/fuel	Estimate (g CO_2_e/kWh)
Wind	2.5 MW, offshore	9
Hydroelectric	3.1 MW, reservoir	10
Wind	1.5 MW, onshore	10
Biogas	Anaerobic digestion	11
Hydroelectric	300 kW, run-of-river	13
Solar thermal	80 MW, parabolic trough	13
Biomass	Forest wood co-combustion with hard coal	14
Biomass	Forest wood steam turbine	22
Biomass	Short rotation forestry co-combustion with hard coal	23
Biomass	Forest wood reciprocating engine	27
Biomass	Waste wood steam turbine	31
Solar PV	Polycrystalline silicone	32
Biomass	Short rotation forestry steam turbine	35
Geothermal	80 MW, hot dry rock	38
Biomass	Short rotation forestry reciprocating engine	41
Nuclear	Various reactor types	66
Natural gas	Various combined cycle turbines	443
Fuel cell	Hydrogen from gas reforming	664
Diesel	Various generator and turbine types	778
Heavy oil	Various generator and turbine types	778
Coal	Various generator types with scrubbing	960
Coal	Various generator types without scrubbing	1050

To find the average CO_2_ emission per kWh of generated electricity, researchers need to have a primary energy use for electricity generation in selected countries and the total electricity usage for selected areas from secondary data then calculate produced CO_2_ based on primary electricity shares for each country or region.

**Fig. 3:**
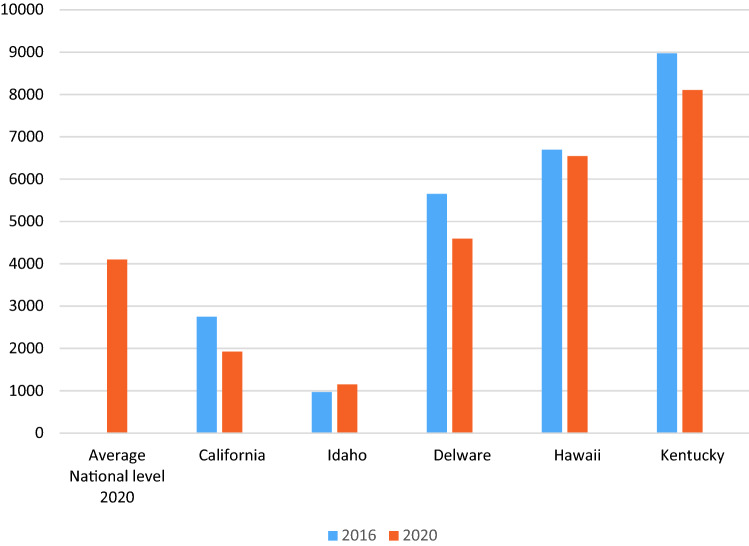
Average EV pollution (2016–2020)

The USA attempted to change the primary energy source from coal to gas between 2010 and 2017. During this period, the country successfully reduced electricity emissions (EESI 2018; Martin and Saikawa 2017). However, a wide gap still exists among the electricity emissions in different states. For instance, in 2020, the released data by Alternative Fuels Data Center (AFDC) confirm that the CO_2_ emitted from electric cars has decreased to 1922 in California, decreased to 972 in Idaho and decreased to 8106 pounds of CO_2_ equivalent (Peters 2020) in Kentucky, making the use of electric cars in the USA more reasonable than before. Figure 3 shows a comparison between EV pollution in national and selected US states between 2016 and 2020 (AFDC 2020). Figure 3 illustrates EV pollution in selected American states and the US average.

Therefore, although the primary sources of energy in different American states vary, the use of EVs in the entire country is still rational and increasing.

‘Fight against Pollution’ is the name of the anti-emission plan of China that was announced by President Xi Jinping in 2014. According to the energy revolution, China planned to move towards clean energy. China, as the world’s largest energy-consuming nation, also announced a reactor plan to accelerate the substitution of low-emission energy (Ahlers and Shen 2018). In addition, China set a plan for cleaning air by giving subsidies to farmers, stopping them from burning straws and relocating polluter factories (Wood 2019).

**Fig. 4:**
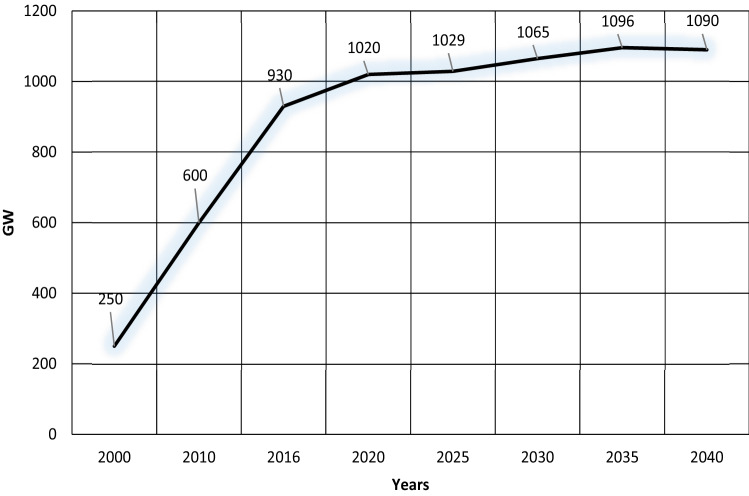
Coal usage in China’s energy sector from 2000 to 2040 IEA-China 2017)

An estimation shows that China’s economy is growing rapidly with an average of 4.5% annually, but because of the combined effects of structural shifts in the economy and strong energy efficiency policies, China’s demand for energy grows only 1% annually (IEA-China 2017). China’s growing energy needs are answered by renewable energies and hydro. However, all these plans and strategies do not mean that China is going to give up burning coal (Qi et al. 2016). Although the share of coal is decreasing in China’s total energy, the usage of coal is increasing from 945 GW in 2016 to 1096 GW in 2035 (IEA-China 2017). Anticipation of coal usage in China’s energy sector from 2000 to 2040 is indicated in Fig. 4.

China is the first producer of solar power in the world, although the share of generated electricity in the total electricity is less than 8%, making China the largest carbon emitter in the world (Gallagher et al. 2019; Xing et al. 2020). Moreover, China’s energy sector attempts to shift from burning coals to clean renewable energies, such as solar. China’s solar plan supports home solar panels and massive solar farms, such as the largest world solar farm in the Tengger Desert (Edmond 2019). However, a new paper published by *Nature Energy* confirms that countries burn a high amount of coal, even blocking the sun’s ray, which can cause an inefficient harvesting of solar energy (Sweerts et al. 2019).

In 2020, China transformed from a country that leans on coal to semi-coal. A positive growth in renewable energy use promoted the carbon emission in China compared with that in the last decade. China, a country that depended on coal, became a semi-coal user in 2020. In the current situation, hydropower has a prominent role in the carbon intensification in the country. Accordingly, states that can generate electricity by water, such as Hubei, Qinghai, Sichuan and Yunnan, can reduce emission until 600 g CO_2_/kWh (Gallagher et al. 2019; Xing et al. 2020). Most states are still producing carbon emission higher than 850 g CO_2_/kWh. At the national level, carbon intensities vary between 861 and 821 g CO_2_/kWh (Xing et al. 2020). Considering the carbon life cycle for petrol and diesel, regular fuel cars in most Chinese provinces make less pollution than EVs. Producing an electric car in China can cause more pollution than in Western countries.

India, as the second most populated country in the world, has a key role in the future energy market. The Indian government has taken many positive steps towards improving public access to electricity for the Indian people. India has also implemented major steps in developing renewable energies, especially solar energy. According to India Power Ministry, the country generated 372K MW electricity in 2020, which is a combination of 53.7% coal, 1.7% lignite, 6.7% gas, 0.1 diesel, 12.3% hydro, 1.8% nuclear and 23.7% renewable energy sources (MoP-India 2020). Attention to the Indian population and the country target makes India worth considering as one of the future leaders of EVs. Although India mainly focuses on two- and three-wheeler EVs, coal remains the major electricity source for electricity in India (Tong et al. 2018). Based on our calculation, CO_2_ emission from electricity generation in India is almost 625 g CO_2_/kWh in 2020, making EV usage and production inept. India pledged to reduce carbon emission up to 33% until 2030 (Timpereley 2020). It will happen by increasing the share of solar and nuclear electricity to 40% of the total country electricity (Kennedy 2015). That is, 1 kWh electricity less than 410 gCO_2_ is expected to be produced in 2030.

### Game theory approach

Authors use game theory as an analytical tool to interpret current EV situations and discuss the insights and our suggestions. The economic application of game theory can be a valuable tool to aid in the fundamental analysis of EV industry, market and any strategic interaction between significant producers or markets.

Game theory, which analyses the welfare-maximising mechanism, can help us better understand how the other incentives can affect social optimism targets. Authors actually employ game theory to check the above argument. By implementing this game and analysing Nash equilibrium, researchers aim to examine the possibilities of countries’ decision. Learning the countries’ preferences in a tight economic and competitive situation is necessary. The voting game (Maksymilian 2017) environment is set up, and the welfare-maximising mechanism is designed to analyse optimal social strategy. The Nash equilibrium indicates that the countries have incentives to use the electricity sources that can provide the lowest electricity cost in production and consumption in EVs.

There are *n* risk-neutral countries (players) in the world, numbered $1,2, \ldots , n.$ Let *N* represent this world, so that

1 $$N = \left\{ {1, \ldots , n} \right\}\;{\text{where}}\;\left| N \right| \ge 2.$$

Authors assume that each country is rational and targets to maximise its utility. The countries have common knowledge of the game and choose their strategies simultaneously. The strategies in this game structure refer to a matrix of two decisions – using resources (clean/unclean electricity sources) for producing EVs and using EVs.

Let $S = \left\{ {s_{1, } s_{2, \ldots , } s_{n} } \right\}$ be a set of strategy where

2 $$s_{i} = \left\{ {s_{ip} , s_{ic} } \right\},\;\forall i \in N.$$

Let ${\Omega } = \left\{ {G,W} \right\}$ be a set of electricity sources. G refers to clean electricity sources and W refers to unclean electricity sources. $s_{ip}$ is country $i$’s strategy/decision of using electricity sources in producing EVs. $s_{ic}$ is country $i$’s strategy/decision of using electricity sources in using EVs. Therefore $s_{i} = \left( {G,W} \right)$ means country $i$ prefers clean electricity sources in production EVs and unclean electricity sources in using EVs.

A country $i$ assigns a value $v_{i}$ on EV market and it remains as public information. Note that a range of industry index and Macroeconomic data can be used to estimate the values. To simplify the analysis, assumes that ${ }v_{i}$ is public information. The value is economy benefit generated by engaging in EV market via production and/or consumption for each country. Thus,

3 $$v_{i} \in \left[ {0, \overline{v}} \right] \;{\text{where}}\; i \in N.$$

Let $G_{i} \left( {v_{i, } v_{ - i} , G} \right)$ be a country $i$’s total electricity cost function of using EVs when $i$ prefers clean electricity sources, given other countries’ benefits of engaging in EV markets. Let $W_{j} \left( {v_{j, } v_{ - j} , W} \right)$ be a country $j$’s total electricity cost function of using EVs when $j$ prefers unclean electricity sources, given other countries’ benefits of engaging in EV markets. The total electricity cost function for country $i$ in using EVs is

4 $$E_{i} \left( {v_{i, } v_{ - i} ,s_{ic} } \right) = kG_{i} \left( {v_{i, } v_{ - i} , s_{ic} } \right) + \delta W_{i} \left( {v_{i, } v_{ - i} , s_{ic} } \right),$$

where

5 $$\left\{ {\begin{array}{*{20}c} {k = 1\;{\text{ and}}\;\beta = 0,} & {{\text{if}}} & {s_{ic} = G, \;\forall i \in N} \\ {k = 0\; {\text{and}}\;\beta = 1,} & {{\text{if}}} & {s_{ic} = W, \forall i \in N} \\ \end{array} } \right..$$

Let $t_{i} \left( {v_{i, } v_{ - i} , S_{p} } \right)$ be the $i$’s total electricity cost function in producing EVs with the property

6 $$\frac{{\partial t_{i} \left( {v_{i, } v_{ - i,} S} \right){ }}}{{\partial v_{i} }} > 0$$

7 $$\frac{{\partial t_{i} \left( {v_{i, } v_{ - i,} S} \right){ }}}{{\partial v_{ - i} }} < 0$$

where $$S_{p} = \left\{ {s_{1p,} s_{2p,} \ldots \ldots s_{np} } \right\}$$.

The electricity carbon life cycle utility function of each country is

8 $$U_{i} \left( {v_{i, } v_{ - i} , S} \right) = v_{i} - t_{i} \left( {v_{i, } v_{ - i} , S_{p} } \right) - E_{i} \left( {v_{i, } v_{ - i} ,s_{ic} } \right),$$

where

$$\forall i \in N; \forall - i \in N;v_{i} \in \left[ {0, \overline{v}} \right]; s_{i} \in S.$$

The participation constraint of each country ensures that they are better off playing in the EV market. That is,

9 $$U_{i} \left( {v_{i, } v_{ - i} , S} \right) > 0, \forall i \in N; \forall - i \in N;v_{i} \in \left[ {0, \overline{v}} \right]; s_{i} \in S.$$

Each country targets to maximise its electricity carbon life cycle utility by choosing strategy $S$. In other words, each country targets to minimise its total electricity carbon life cycle cost by choosing which electricity sources to use in production and consumption. That is,

10 $$\min t_{i} \left( {v_{i, } v_{ - i} , S_{p} } \right) + E_{i} \left( {v_{i, } v_{ - i} ,s_{ic} } \right)\;{\text{where}}\;\forall i \in N; \;\forall - i \in N;v_{i} \in \left[ {0, \overline{v}} \right];\; s_{i} \in S$$

subject to

11 $$U_{i} \left( {v_{i, } v_{ - i} , S} \right) > 0$$

12 $$\frac{{\partial t_{i} \left( {v_{i, } v_{ - i,} S} \right){ }}}{{\partial v_{i} }} > 0$$

13 $$\frac{{\partial t_{i} \left( {v_{i, } v_{ - i,} S} \right){ }}}{{\partial v_{ - i} }} < 0.$$

The Nash equilibrium for country $i$ is (G, G) if

14 $$t_{i} \left( {v_{i, } v_{ - i} , G, s_{ - ip} } \right) < t_{i} \left( {v_{i, } v_{ - i} , W, s_{ - ip} } \right)\;{\text{and}}\;G_{i} \left( {v_{i, } v_{ - i} , G} \right) < W_{i} \left( {v_{i, } v_{ - i} , W} \right).$$

The electricity carbon life cycle utility-maximising analysis indicates two behaviours. Firstly, all countries prefer the electricity sources that can offer the lowest total electricity cost in production and consumption of EVs. Secondly, the countries with comparative lower electricity cost (assigns higher value) in EV production will reduce the total electricity cost of the countries with comparatively higher electricity cost in EV production. Alternatively, it indicates a possibility that the countries with unclean electricity sources have cost leadership in the EV market and become the large EV exporters.

The social optimisation (environmental perspective) aims to maximise all countries’ utility and reduce CO_2_ emission worldwide. In other words, social optimisation targets to minimise the total electricity cost of production and use EVs, subject to the condition that this EV market project can reduce global warming or CO_2_ emission worldwide. That is,

15 $$min\mathop \sum \limits_{i \in N} t_{i} \left( {v_{i, } v_{ - i} , S_{p} } \right) - \mathop \sum \limits_{i \in N} E_{i} \left( {v_{i, } v_{ - i} ,s_{ic} } \right),$$

subject to

16 $$\frac{{\partial g\left( {v_{i, } v_{ - i, } S} \right)}}{{\partial v_{i} }} \le o,\;\forall i \in N,$$

where $g\left( {v_{i, } v_{ - i, } S} \right)$ is the CO_2_ emission function.

To achieve the reduced CO_2_ emission target in this social optimisation problem, clean electricity sources are required in the production and consumption of EVs used by all the players in this game. The players (countries) are suggested to improve the capacity of generating electricity with clean sources, that is, to reduce $G_{i}$ for any level of sources, so that $G_{i} \left( {v_{i, } v_{ - i} , G} \right) < W_{i} \left( {v_{i, } v_{ - i} , W} \right)$. The countries with unclean electricity sources in production and consumption EVs can revise their plan to increase the incentive of using clean electricity sources in the first instance.

The welfare-maximising mechanism finds that the individual utility-maximising behaviour deviates from social optimisation. Countries using unclean electricity sources with a lower total electricity generating cost may have cost leadership in the EV market and become the large exporters. Moving the Nash equilibrium to social optimisation, the authors suggest the universal agreement of minimising the number of countries using unclean electricity sources in the production and consumption of EVs. The purpose of this proposal is not to exempt or demotivate some countries engaged in the EV market. Instead, it encourages the countries to invest in infrastructures so as to improve the capacity of electricity generating using clean sources. Welfare maximisation can then be achieved dynamically.

## Discussion

Considering that the electricity market is not based on production cost, if electricity is generated from renewable and clean sources, then it becomes more expensive than usual. People will naturally lose interest in EVs, considering that fossil fuel is a cheap energy resource. This condition holds true, given that having cheap clean energy in many countries remains impossible. In addition, the instabilities of oil prices and oil supply are other reasons countries are generally interested in accelerating the EV market. The instabilities in the Middle East and Ukraine make these countries be concerned about the future of the oil and gas market (EFA 2022). All universal declarations and agendas urge countries to reduce carbon emissions and be involved in the fight against global warming (Jakob 2020). The countries of some of the largest energy producers are less interested in following universal coal restriction rules.

China, the USA and India are the top coal producers in the world (Dillinger 2020). Coincidently, China and the USA have the largest EV markets in the world (IEA 2020), and India has major future plans (BBC 2019) for EVs. Fluctuations in oil prices and expensive renewable energies impair many countries. Countries that do not have cheap energy sources are willing to accept, discuss and implement clean energy, whereas those with access to unlimited cheap coal or oil are not interested in clean energy. They do not see any economic advantage in renewable energy. They have considerable fuel resources and, clearly, they cannot simply dispose of all coal sources, close the mines or stop oil refining because universal declarations do not allow them to use these sources.

Based on the CO_2_ emission per kWh mentioned in the literature and the EV production in major electric car markets, authors can certainly say that European EVs are not air polluters. In Europe, EVs can help with anti-global warming initiatives if they are used instead of petrol cars.

In this situation, world organisations must observe whether China and India will continue setting the ambitious target as before or will be involved in an economic competition. Researchers should consider that China, as the owner of the largest coal mines in the world, has access to the cheapest sources of energy, which always motivates their use in power plants (Fan et al. 2020). Similar to countries in the EU, China, India and the USA, other countries have major future plans for developing EVs. Many of these countries have already set different types of incentives to support the demand and supply of electric cars (Gong et al. 2020). Most of these countries still generate high emission electricity, which are the main sources of their EVs. Generating electricity in India, Australia, South Africa, Greece, Malaysia and other countries producing high amounts of CO_2_ and involving each of these countries in EV development will increase universal carbon emissions more than before (Xue et al. 2021).

Accordingly, if a country or state is generating electricity with an average pollution less than petrol or diesel (less than 700 g CO_2_/kWh), then we should encourage governors to develop EV usage; otherwise, the main policy should focus on decreasing electricity CO_2_ emission. For producing EVs in factory, countries must have low electricity emission because producing electric cars causes more pollution than producing other cars (approximately 600 g CO_2_/kWh). Considering electricity drop in grid connection can reduce this amount.

This study highlights the issues that the level of carbon dioxide production of electric cars is not limited to daily electricity consumption and that it is not the same in all part of the world. In the calculations and policies, extra produced carbon in EV production should be considered, too. After all, the goal of universal declarations and policies should be reducing the level of carbon dioxide and not increasing the production of electric cars. In this regard, paying attention to the resources of the generated electricity in each country and region can be the main discussion of any declaration’s draft.

Nash equilibrium in game theory confirms a possibility that the countries using unclean electricity sources with a lower total electricity generating cost have cost leadership in the EV market and become the large exporters. The application of game theory in this study also shows that the significant EV producers and markets have incentives to use the electricity sources that can provide the lowest electricity cost in production.

Accordingly, in universal declarations, the priority must be given to primary electricity sources. Developing EV production with the current primary sources in China, India and some developing countries generates more pollution than current fossil cars. Although carbon emission and global warming are universal problems, the regional policies following by local approaches, in the case of EVs, can sometimes be more efficient than universal policies. This study proposes that the first target of universal declarations and agreements must aim for the infrastructure of each country instead of sectorial planning. That is, a country, which is still generating electricity with pollution higher than 550 g CO_2_/kWh, should prioritise the maintenance of energy sources rather than the production and development of EVs.

In answer to the question of this study; naturally, all countries in the world are interested in a better place to live in, and governments agree that a better environment can be realised by producing fewer pollutants. However, fluctuating oil prices owing to political tensions and economic competitions on the one hand and the high price of renewable energies on the other hand have made it very difficult for countries to entirely and suddenly stop consuming coal, which is too cheap for them. Moreover, many jobs and local economic growth and development in these countries continue to depend on coal mining. The non-production of environmental pollutants is not related to EV production but the change of economic infrastructure and generation of clean energy.

A notable limitation of this study is the carbon emission data in power plants. Usually, these figures are an estimation based on energy usage and capacities in each country. By contrast, in the pick of electricity usage, the emitted CO_2_ is much higher than the given figures. Factors such as lack of wind or reduced sunny days in European countries also force some of these countries to use fossil fuels temporarily to address electricity shortages.

Another limitation of this study is the lack of accurate statistics on carbon emissions during EV production. Despite the possibility of calculating this figure, owing to the highly competitive condition of the car production market, the exact carbon figures are not published by the manufacturers. The publishing of these statistics can be considered a negative point by manufacturers and can even disrupt the production of electric cars. Therefore, the statistics provided in this regard are not accurate statistics by automakers but a variety of estimates provided by researchers. In many cases, the material of the engine, body and battery are secrets of the manufacturers, which makes even the estimates impossible. In addition, the rapid change in the industry in terms of engine and body and the rapid evolution of batteries have made accessing accurate data on pollutants in the EV production challenging.

Electric cars are one of the main components of reducing carbon emissions. The policies of international organisations to reduce emissions through the development of electric vehicles globally have shown their effectiveness in European countries. As the world’s largest economies with a significant share in the production of pollutants, China and India have written their plans and announced them to the world. These countries and even other developing countries only need time to invest in producing low-emission energy to join the fight to reduce carbon emissions and global warming. Countries must accelerate EV production with low-emission energy production to help the universal movement in this battle.

## Conclusion

Western countries, along with China and India, have started comprehensive and codified programmes for the development of EVs. However, the study indicates that some countries producing EVs have not yet reached the ideal limit for low-carbon electricity generation.

The proposed regulation suggests countries not to be involved in the economic competition in the EV sector as they must prioritise electricity as the largest source of emissions. Otherwise, the current approach by universal declarations is shunting investments towards final production instead of investing in infrastructures, such as renewable energy or clean resources for power plants.

In some cases, developing EV production with the current primary sources in China, India and some developing countries generates more pollution than current fossil cars. Although carbon emission and global warming are universal problems, the regional policies following by local approaches, in the case of EVs, can sometimes be more efficient than universal policies. This study proposes that the first target of universal declarations and agreements must aim for the infrastructure of each country instead of sectorial planning. That is, a country, which is still generating electricity with pollution higher than 550 g CO_2_/kWh, should prioritise the maintenance of energy sources rather than the production and development of EVs.

## Data Availability

Enquiries about data availability should be directed to the authors.
